# Supplementary figures and images for: Generation of a bloodstream form Trypanosoma brucei double glycosyltransferase null mutant competent in receptor-mediated endocytosis of transferrin
Source: PLoS Pathog. 2024 Jun 27;20(6):e1012333. doi: 10.1371/journal.ppat.1012333 (PMC11236118; doi:10.1371/journal.ppat.1012333)

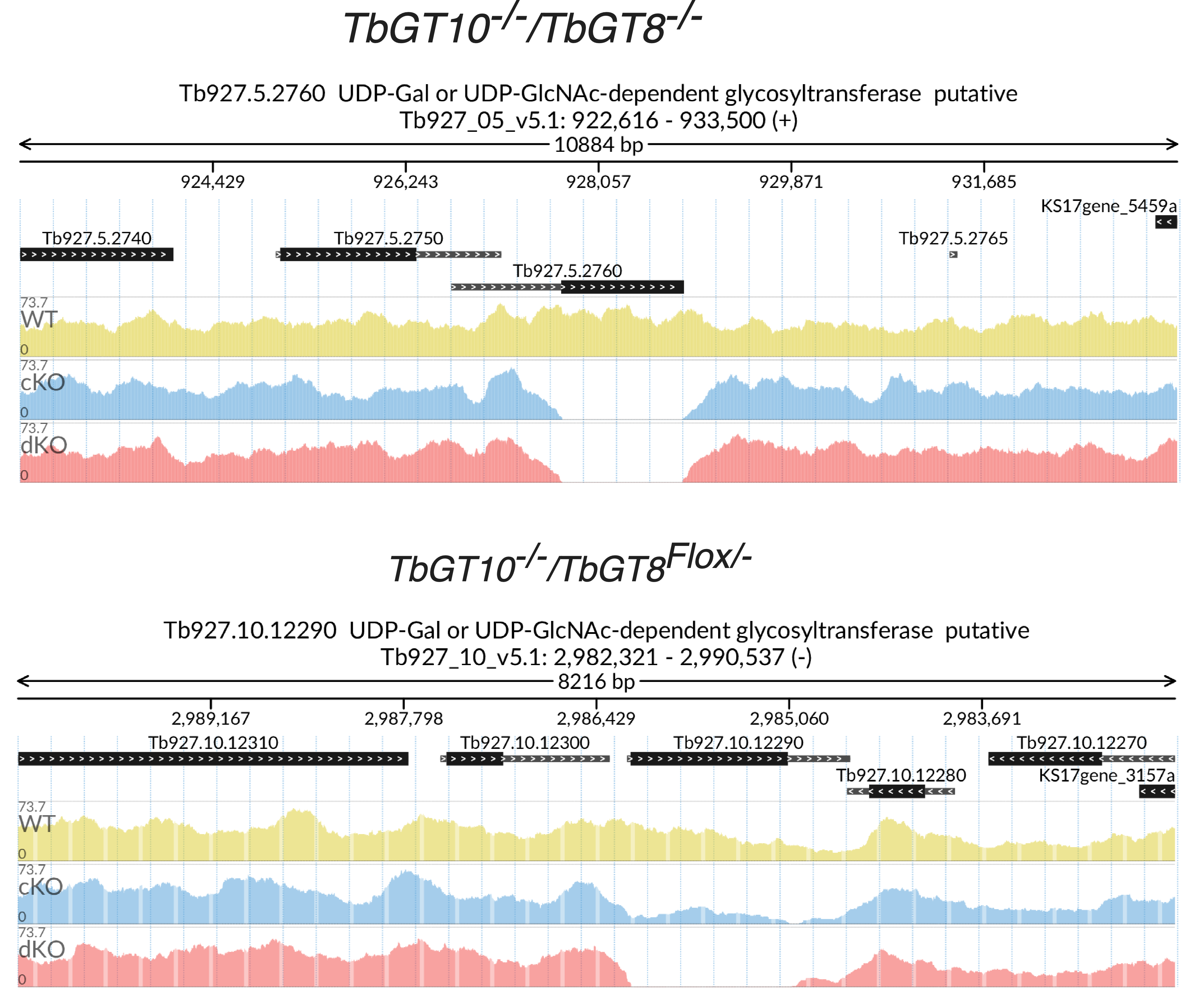

Supplement: S1 Fig — Genomic DNA harvested from WT, TbGT10-/-/TbGT8Flox/- conditional null mutant (cKO) and TbGT10-/-/TbGT8-/- double null mutant (dKO) cells was subjected to whole genome sequencing (30x coverage, paired end reads) and aligned to the Trypanosoma brucei brucei TREU927 reference genome. The plot shows mapped reads at the target genomic loci for TbGT10 (Tb927.5.2760) and TbGT8 (Tb927.10.12990). (TIF) [file ppat.1012333.s001.tif]

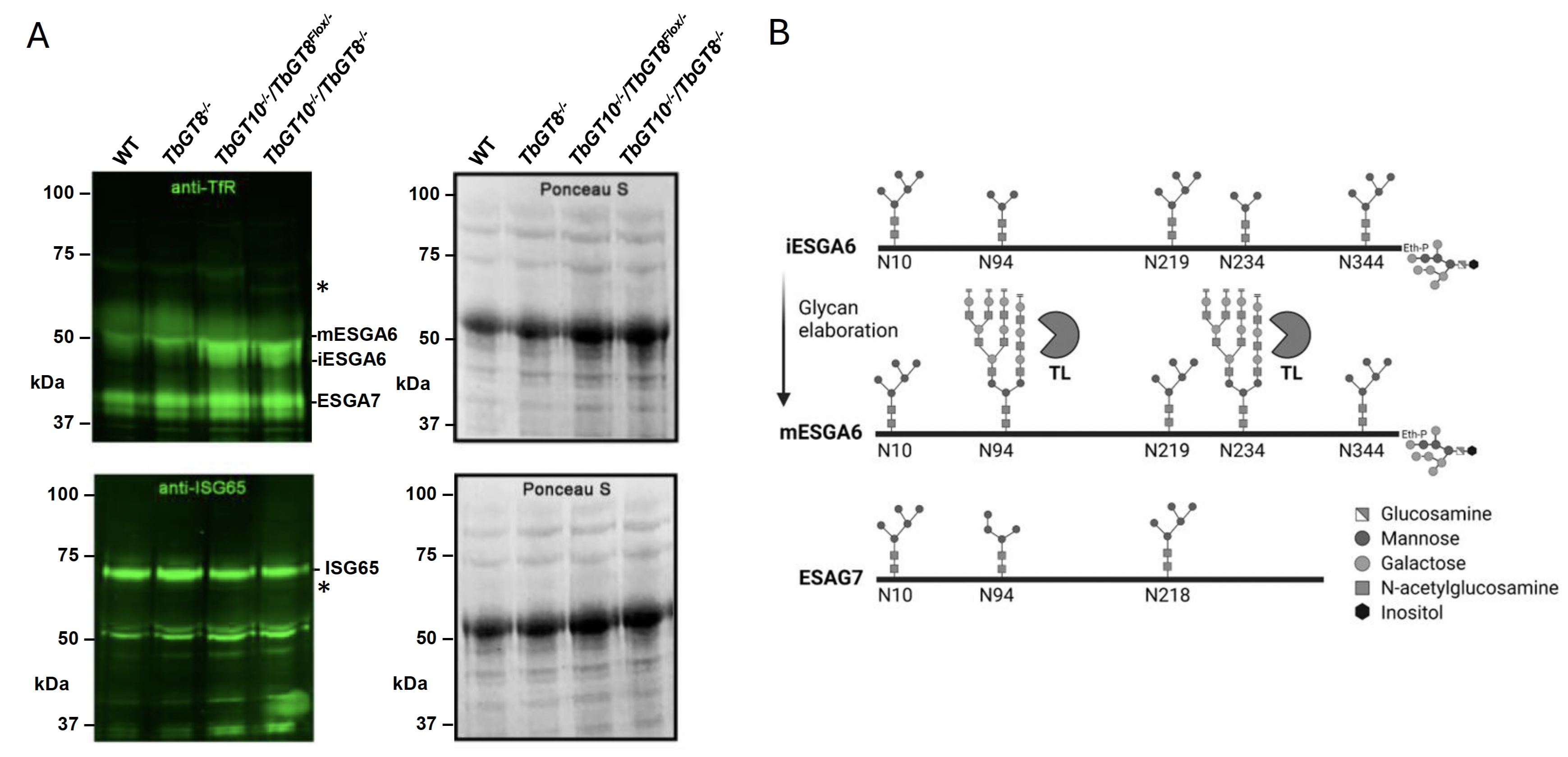

Supplement: S2 Fig — Cell ghosts were purified from soluble VSG (sVSG) by osmotic lysis. sVSG from WT, TbGT8-/-null mutant, TbGT10-/-/TbGT8Flox/- conditional null mutant and TbGT10-/-/TbGT8-/- double null mutant cells were resolved by SDS-PAGE and transferred to nitrocellulose. Western blotting (green) with anti-transferrin receptor (TfR: upper panels) detecting both expression site associated genes (ESAG) 6 and 7 and anti-invariant surface glycoprotein 65 (ISG65: lower panels). Equal loading and transfer are demonstrated by Ponceau S staining (right). The region corresponding to the RCA reactive band is indicated (*) on the right and the molecular weight markers shown on the left. Products corresponding to mature (m)ESAG6, immature (i)ESAG6 and ESAG7 are indicated on the right. B. A schematic displaying the N-linked glycosylation states of immature and mature ESGA6 and ESAG7. Mature ESAG6 is reactive with tomato lectin (TL). (TIF) [file ppat.1012333.s002.tif]

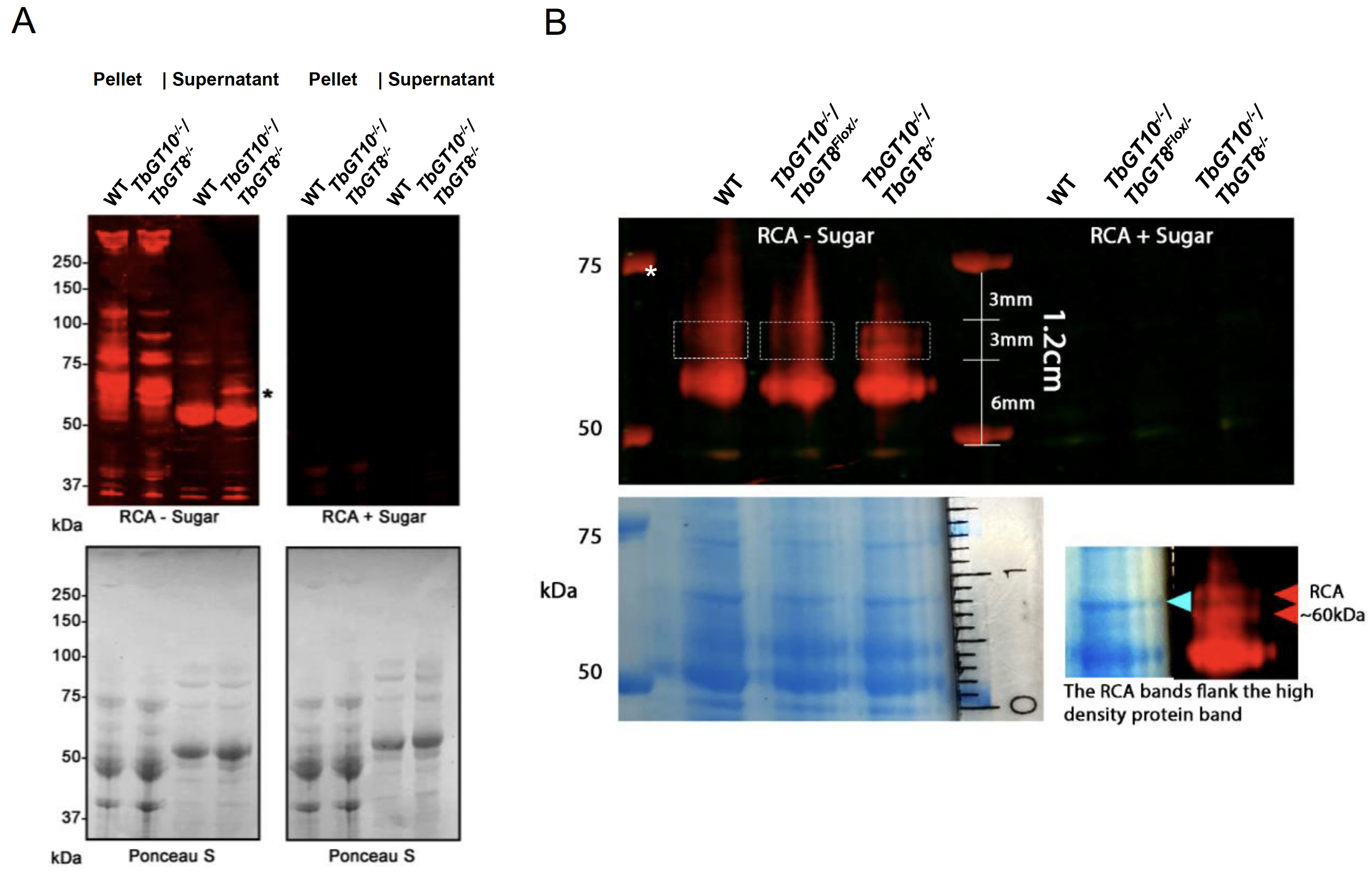

Supplement: S3 Fig — Cell ghosts (pellet) were purified from VSG fraction (supernatant) by osmotic lysis from WT and TbGT10-/-/TbGT8-/- double null mutant cells. Pellet and supernatant fractions were resolved by SDS-PAGE and transferred to nitrocellulose in duplicate. Membranes were incubated with biotinylated RCA (red) without (RCA–Sugar: left) or with (RCA + Sugar: right) pre-incubation with 30 mg/ml galactose and lactose. Equal loading and sample transfer are demonstrated by Ponceau S staining. B. VSG (supernatant) fractions purified from WT, TbGT10-/-/TbGT8Flox/- conditional null mutant and TbGT10-/-/TbGT8-/- double null mutant mutants were depleted with anti-VSG221 conjugated agarose beads to remove VSG221 and non-bound material was incubated with RCA lectin agarose beads to purify RCA reactive glycoproteins. RCA agarose bound material was eluted by heating in SDS-sample buffer and resolved in triplicate lanes to enable RCA lectin blotting (upper panel) with RCA pre-incubated with 30 mg/mL galactose and lactose (RCA + Sugar) or not (RCA -Sugar) to enable Coomassie staining (lower panel). Gel slices from each sample corresponding to the RCA doublet were excised and submitted for proteomic analysis. The identified proteins are shown in Table 1. (TIF) [file ppat.1012333.s003.tif]

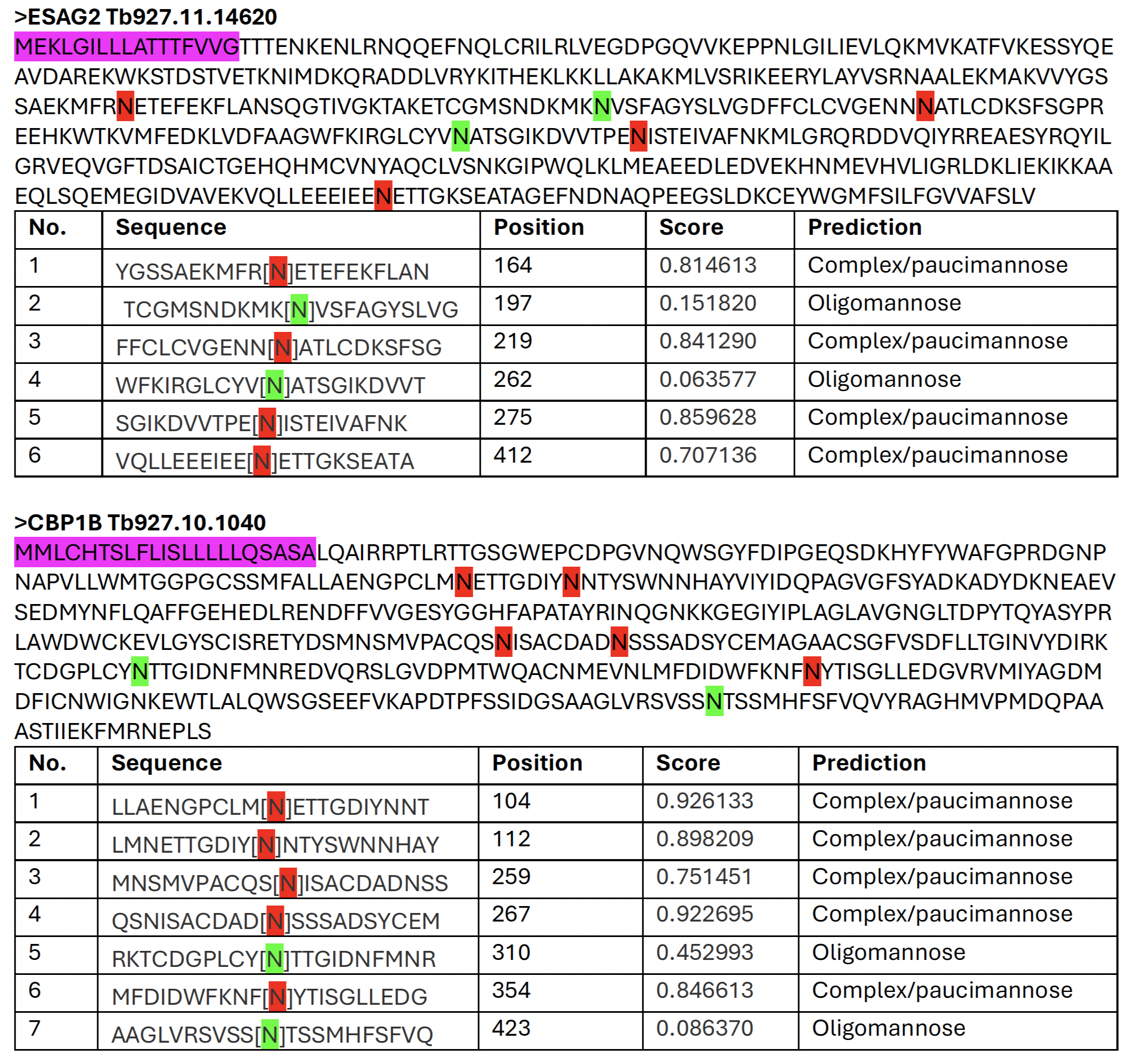

Supplement: S4 Fig — The amino acid sequences from each protein were analysed using the N-glycosylation site prediction software [27]. Complex/paucimannose (red) and Oligomannose (green) glycosylation sites are indicated in the tables alongside their prediction scores and on the amino acid sequences. Signal peptides predicted by SignalP analysis are represented on the amino acid sequences in pink. (TIF) [file ppat.1012333.s004.tif]

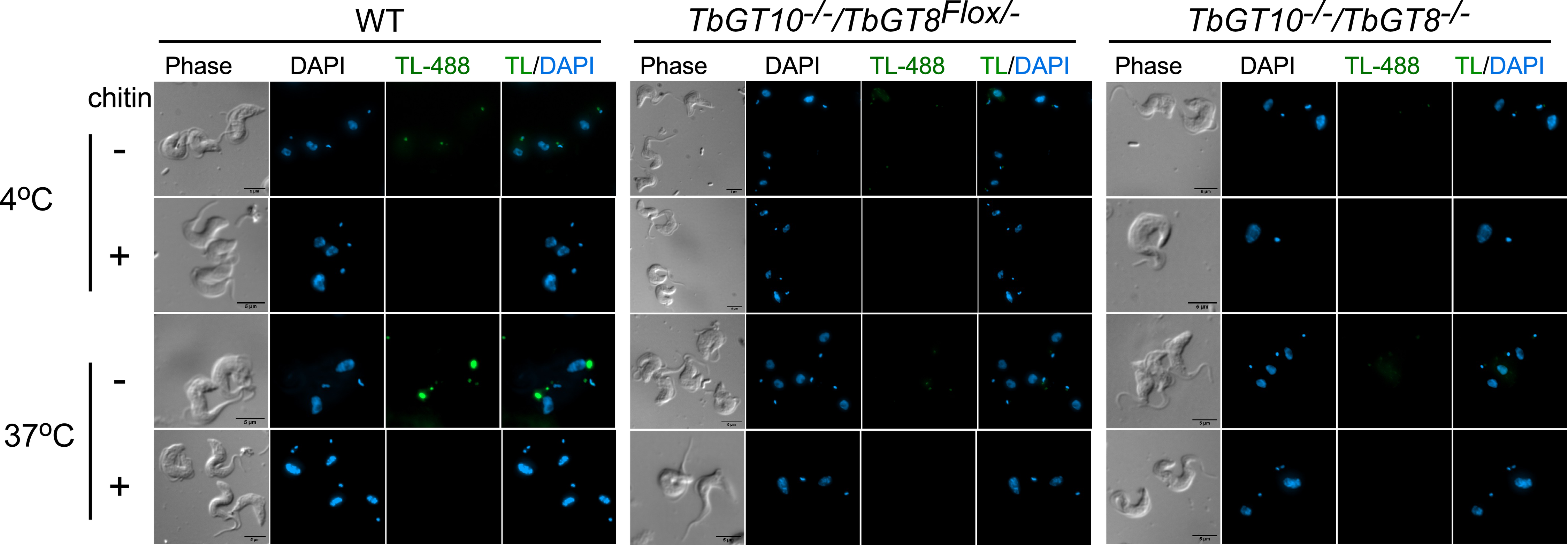

Supplement: S5 Fig — Binding and internalisation of TL were assessed by incubating cells with TL::Dylight488 (TL:488) at 4°C for 5 minutes and then transferred to 37°C for 10 minutes to activate endocytosis in the presence (+) or absence (-) of chitin hydrolysate, as described in Fig 5. Endocytosis was terminated on ice, and half of the cells were processed for flow cytometry (Fig 5B) while the other half were fixed with 2% paraformaldehyde, counterstained with DAPI for nuclei and kinetoplasts, mounted on slides, and imaged by fluorescence microscopy. The flagellar pocket is located adjacent to the kinetoplast, while endocytic compartments are situated between the central nucleus and the kinetoplast, indicated by large (nucleus) and small (kinetoplast) DAPI signals. Scale bar (black line): 5 μM. (TIF) [file ppat.1012333.s005.tif]

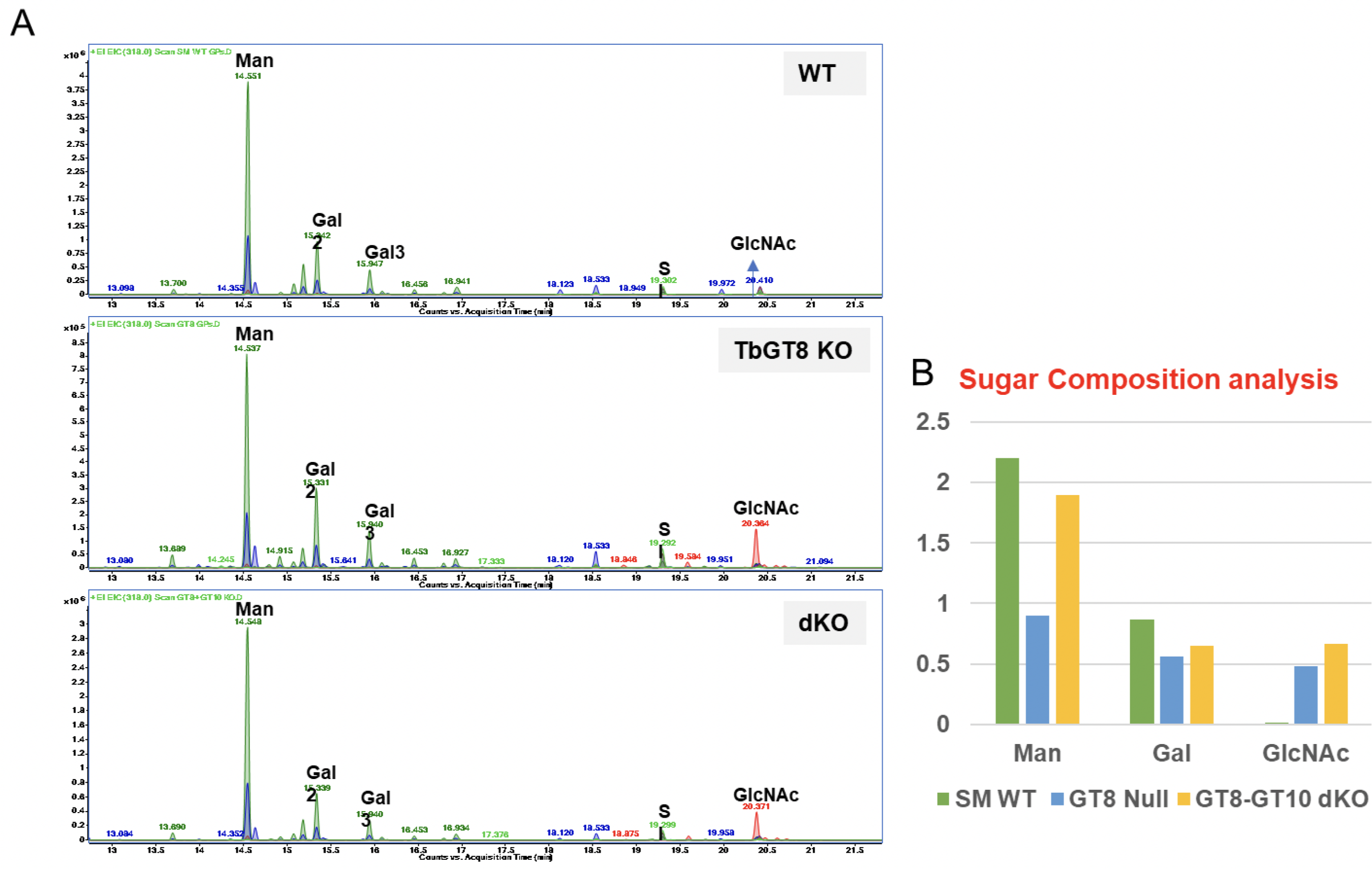

Supplement: S6 Fig — A. The extracted N-glycopeptides were subjected to methanolysis and trimethylsilylation and the obtained methyl glycosides were analysed by GC-MS. 1 nmole of scyllo-inositol was used as an internal standard. B. Abundance of different monosaccharides plotted as a bar graph. Man: mannose, Gal: Galactose, GlcNAc: N-acetylglucosamine. (TIF) [file ppat.1012333.s006.tif]

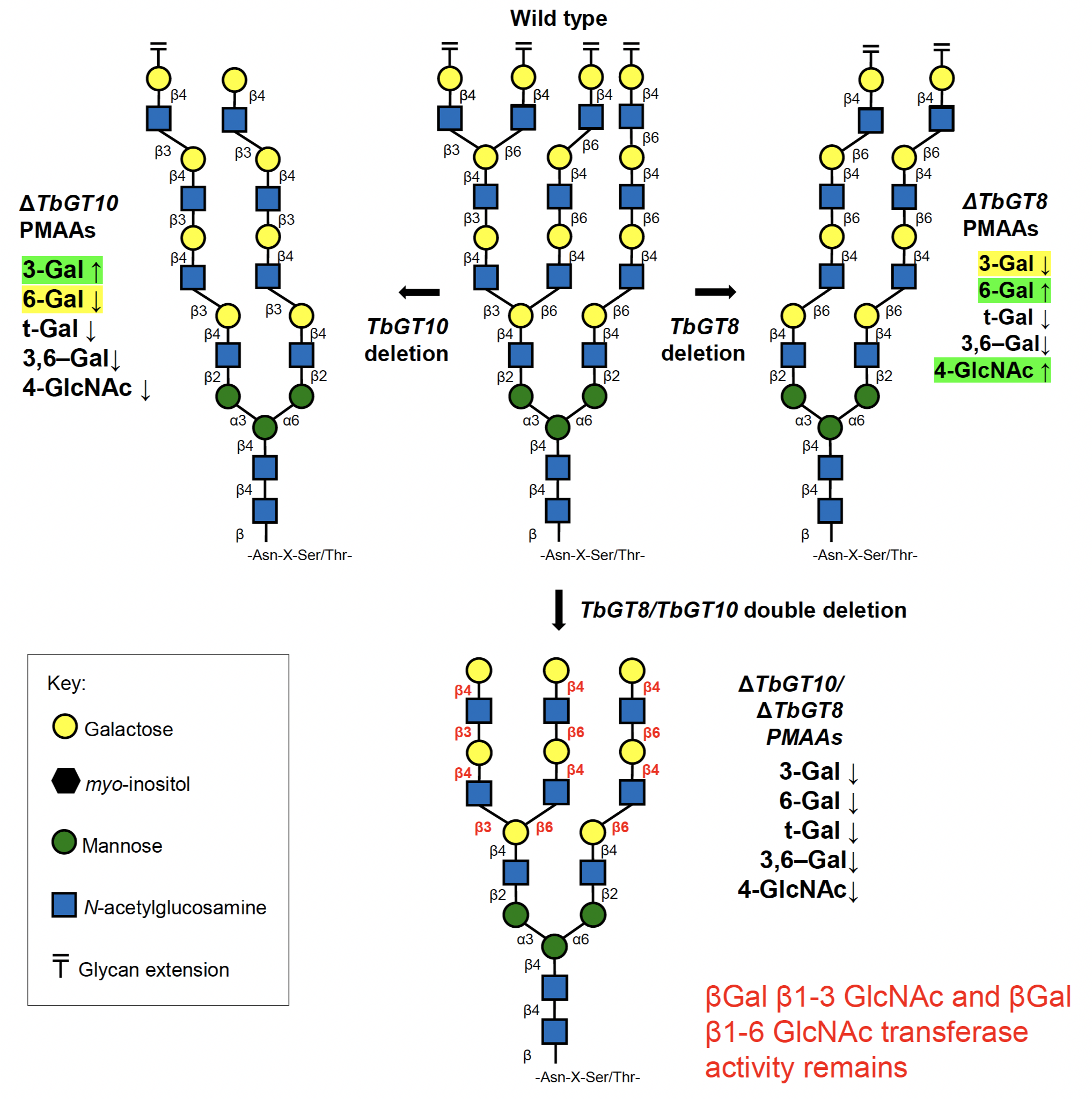

Supplement: S7 Fig — Representative schematic of the complex N-glycan structures synthesised in cells depleted of TbGT10, TbGT8 or both (double deletion), based on permethylation linkage analysis. (TIF) [file ppat.1012333.s007.tif]

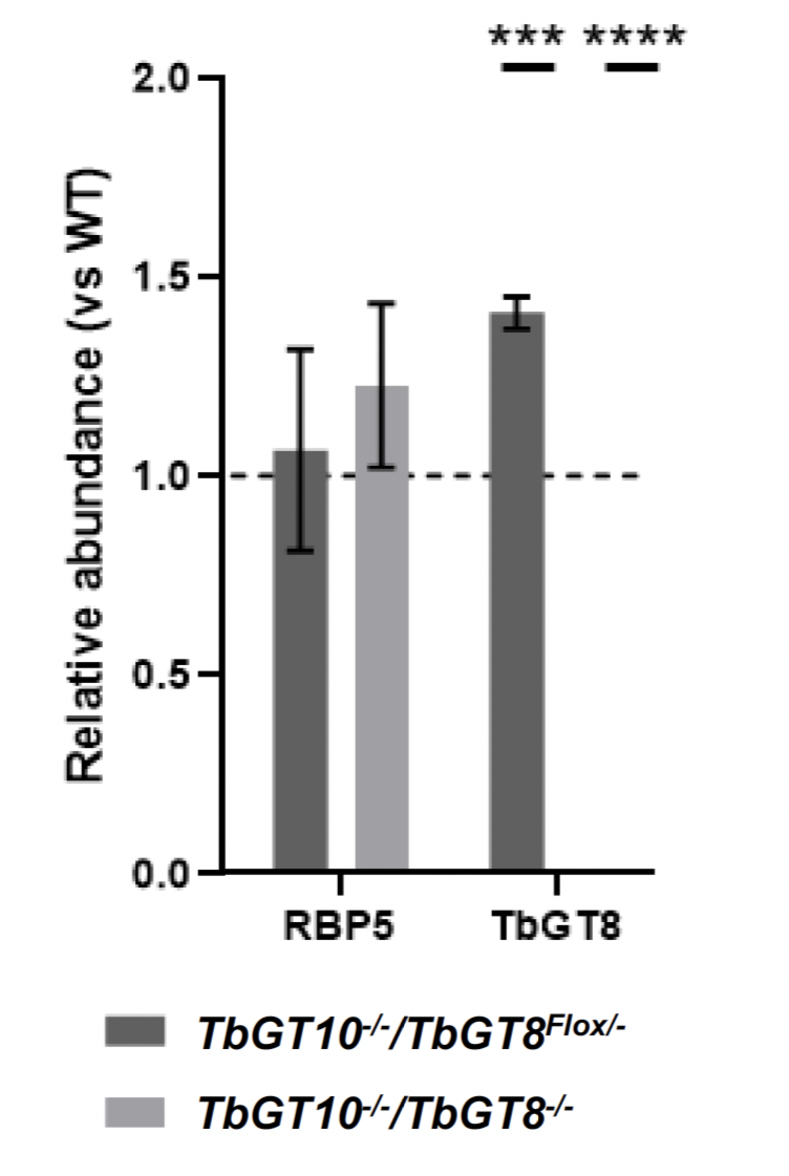

Supplement: S8 Fig — qRT-PCR analysis was performed to investigate RBP5 and TbGT8 transcript abundance in TbGT10-/-/TbGT8Flox/- conditional null mutant and TbGT10-/-/TbGT8-/- double null mutant cells, relative to WT (dashed line). Data are means ± SD (n = 3 biological replicates with 3 technical replicates per n) ***P <0.0005 ****P <0.000005 as determined by unpaired T-test as compared with WT RNA expression levels. (TIF) [file ppat.1012333.s008.tif]

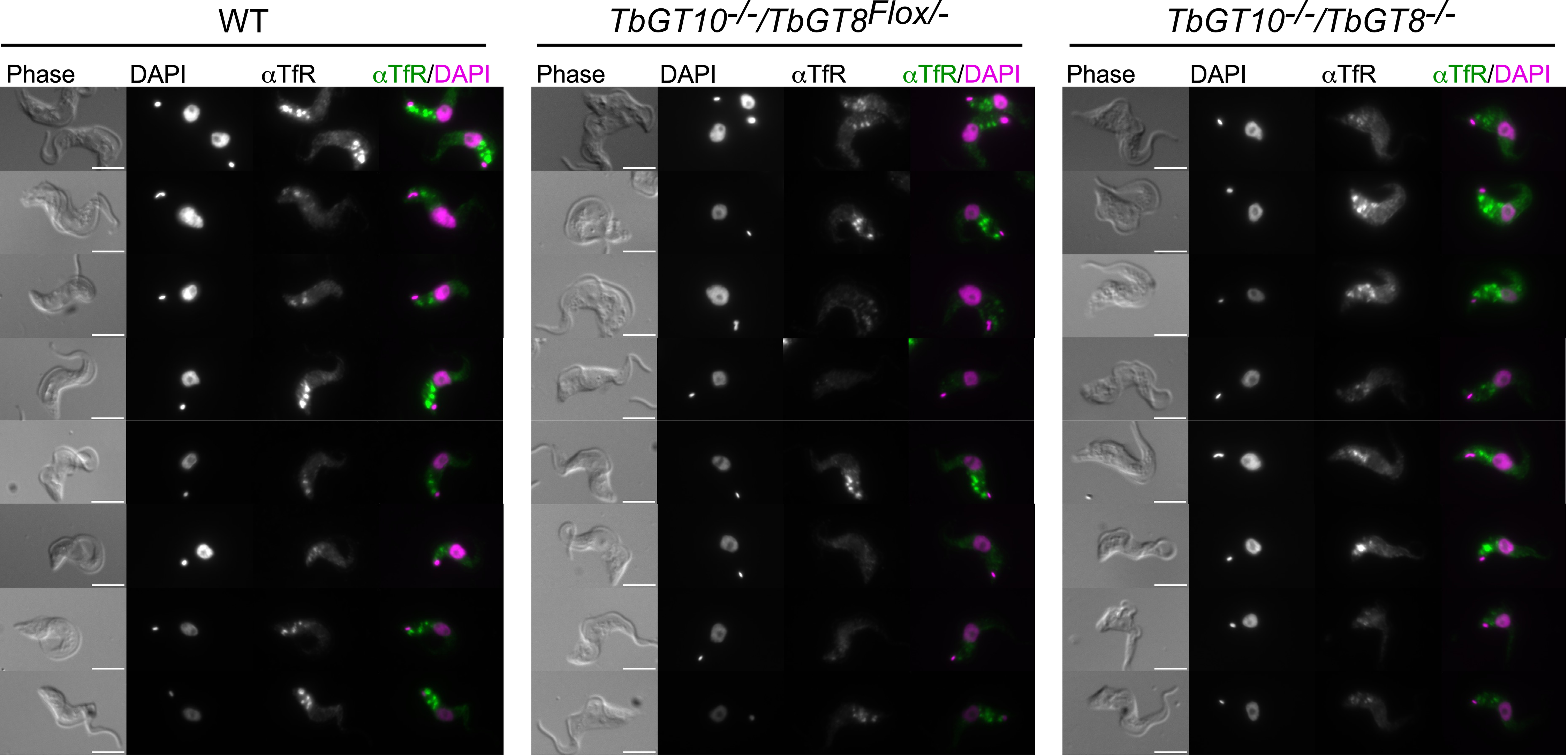

Supplement: S9 Fig — Microscopy was performed on formaldehyde fixed, permeabilised cells stained with DAPI (magenta) and anti-TfR (αTfR, green). Representative cells are presented to show heterogeneity in TfR localisation between the cell lines. Arrowhead indicates flagellar pocket localisation of TfR. Scale bar = 5 μM. (TIF) [file ppat.1012333.s009.tif]

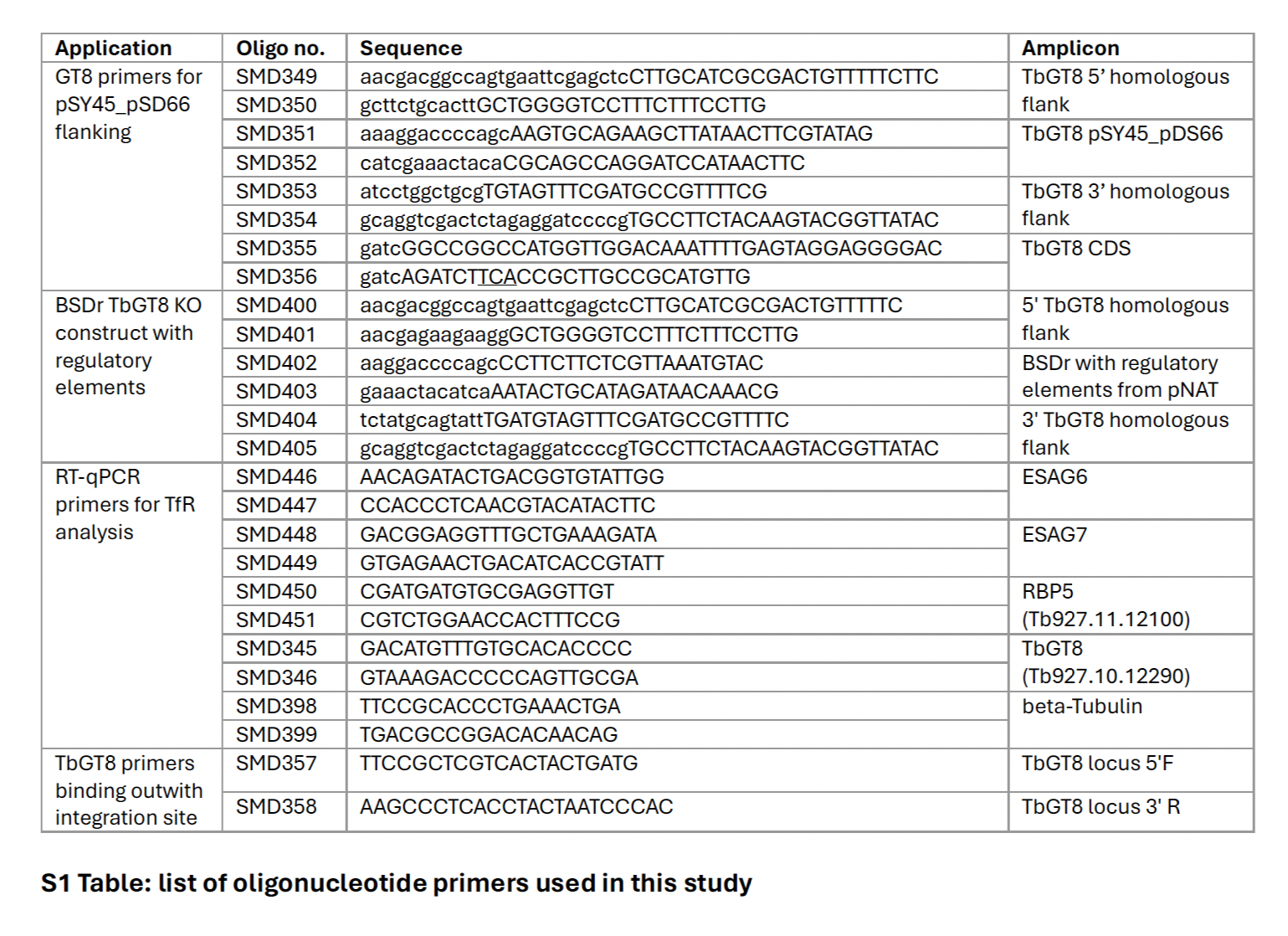

Supplement: S1 Table — (TIF) [file ppat.1012333.s010.tif]
