# Supplementary material for: Generation of a bloodstream form Trypanosoma brucei double glycosyltransferase null mutant competent in receptor-mediated endocytosis of transferrin
Source: PLoS Pathog. 2024 Jun 27;20(6):e1012333. doi: 10.1371/journal.ppat.1012333 (PMC11236118; doi:10.1371/journal.ppat.1012333)
Supplement: Attachment — Submitted filename: Author response - PPATHOGENS-D-24-00742.pdf [file ppat.1012333.s011.pdf]

30 May 2024

Author responses to reviewer comments (Duncan et al, 2024).

Dear Prof. He,

Thank you for handling the review of our manuscript. We genuinely appreciate the constructive feedback and thorough review of our manuscript.

We are grateful to all three reviewers for their thoughtful summaries of our work.

Below, we provide detailed point-by-point responses to each of the reviewers' comments (**black**), our responses (**blue**) and highlight the changes made (**red**) in the revised manuscript. The line numbers in our response refer to those in the revised version of the manuscript.

We believe these revisions have addressed all the reviewers' comments and have significantly strengthened our manuscript. We appreciate the time and effort the reviewers have invested in evaluating our work.

Thank you for considering our revised manuscript.

Part II – Major Issues: Key Experiments Required for Acceptance
Please use this section to detail the key new experiments or modifications of existing experiments that should be absolutely required to validate study conclusions.

Generally, there should be no more than 3 such required experiments or major modifications for a "Major Revision" recommendation. If more than 3 experiments are necessary to validate the study conclusions, then you are encouraged to recommend "Reject".

Reviewer #1:

Comment: I have two related concerns that would improve the manuscript:

1. One of the most striking results is the specific inhibition of TL-488 in Figure 4B/C. It is presumed that the endocytosis is restored at the 14 and 37 C temperatures, but considering how striking the phenotype is I think it would be useful to confirm that the TL-488 is remaining confined to the flagellar pocket in the two mutants. Also, why did the authors choose to display median fluorescence intensity in 4C? In my experience, mean fluorescence of the population is the more commonly used measurement.

Response:
We believe the reviewer is referring to Fig 5B/C. We had performed experiments to confirm the localisation of TL-488, and the data showed that TL::488 largely remains confined to the

flagellar pocket at 37°C in the mutants, as predicted by the reviewer. Initially, we presented only flow cytometry data because it provides an unbiased and more sensitive approach to quantifying fluorescence signal intensity across approximately 10,000 single cells at the population level. For completeness and clarity, we have now included representative fluorescence images of TL binding and uptake at 4°C and 37°C in the supplementary material of the revised manuscript (S5 fig), and added the following text in lines 292-297:

To further confirm the reductions in TL:488 binding and uptake, WT and mutant cells were assessed for their capacity to efficiently endocytose by fluorescence microscopy. The results showed reduced TL signal intensity adjacent to the kinetoplast (flagellar pocket) in the mutants relative to WT cells at 4°C, with minimal or undetectable signal in endocytic compartments (between the nucleus and the kinetoplast) at 37°C when using wild-type TL::488 fluorescence detection limits (S5 Fig).

Regarding the use of median versus mean fluorescence intensities in the flow cytometry data, we chose to display the median fluorescence intensity values because they are more robust to outliers and better represent the central tendency of non-Gaussian data distributions commonly seen in flow cytometry. As suggested by the reviewer, we have recalculated the flow data using mean fluorescence intensity values. This recalculation slightly alters the observed reductions in TL binding at 4°C, where *TbGT10*$^{-/-}$*/TbGT8*$^{Flox/-}$ cells show an increase, contrasting with the previously observed reduction in Fig 5A. Nevertheless, when using the mean intensity values, our overall conclusion that the TL endocytic capacity of *TbGT10*$^{-/-}$*/TbGT8*$^{-/-}$ cells is significantly impaired remains valid, as significant reductions are still observed at 14°C and 37°C. Therefore, we would like to maintain the use of median values as they provide a clearer representation of the central tendency, in agreement with Fig 5A and the new supplementary fluorescence images (S5 Fig).

Comment:

2. In Figure 8, I feel that the data in Figure D and E are somewhat contradictory. The number of Tf binding sites is not compromised, and perhaps seems somewhat elevated in knockouts. However, in E, assuming that equivalent lookup tables were used for the anti-TfR channel, it appears that there is a significant decline in TfR in the pocket region of the cells. From the data in 8A, it appears that there is not a decline in the total amount of TfR in the knockouts, so is it possible that the protein is not being effectively retained in the pocket? Otherwise, it seems that there is less TfR in the pocket of the mutants, but that it provides the same Tf binding capacity at in the WT? A more careful accounting of the TfR microscopy data may provide insight. Is there any TfR present on the cell surface in the KOs?

Response:
We apologise for any confusion regarding TfR localisation. Our data indicate that TfR is retained in the flagellar pocket and not mis-localised to the cell surface in the mutants. We mentioned the slight reduction in the flagellar pocket signal (lines 415-417) and showed two representative cells (Fig 8E) to demonstrate heterogeneity in the anti-TfR signal across the WT, *TbGT10*$^{-/-}$*/TbGT8*$^{Flox/-}$ and *TbGT10*$^{-/-}$*/TbGT8*$^{-/-}$ mutant cell populations, rather than a decrease in total TfR. This heterogeneity does not significantly affect the receptor's ability to bind and endocytose transferrin (Tf), as demonstrated in Figures 8B and 8C. Furthermore, it

does not impact the total number of Tf binding sites at the surface of TbGT10, TbGNTI, or TbGNTII mutants, where pNAL synthesis is disrupted, compared to WT cells, as supported by our flow cytometry assays in Figure 8D.

To further address this apparent discrepancy, we have included more representative images showing this heterogeneity, where some WT and mutant cells were brightly stained with anti-TfR, and others had less fluorescence signal in the flagellar pocket (S9 Fig). This is also partly the reason we adopted the use of median values in our flow cytometry analysis of both TL (Fig 5) and Tf uptake (Fig 8) as a more reliable measure of central tendency to mitigate the effects of outliers. We hope that the additional supplementary figures clarify these points and ensure consistent analysis across the images, allowing readers to make their own unbiased assessments.

Reviewer #2: There are no significant points to address.

Reviewer #3: None

Part III – Minor Issues: Editorial and Data Presentation Modifications
Please use this section for editorial suggestions as well as relatively minor modifications of existing data that would enhance clarity.

Reviewer #1:

Comment: I was a little surprised that ESAG6 is modified with pNAL but does not show as large a MW shift as the other pNAL-modified proteins that are discussed, which have 50-60 kDa attributable to the glycans. Is this due to fewer overall modified N-linked glycans or shorter pNAL repeats? Could this alter the function of the pNAL?

Response:
The difference in the MW shift for ESAG6, compared to other pNAL-modified proteins, is due to a smaller overall number of paucimannose-modified *N*-linked glycans. ESAG6 has two *N*-linked glycans that can be elaborated into complex N-glycans. In contrast, other glycoproteins such as p67, ESAG2, or CBP1B contain 4 to 5 *N*-linked paucimannose glycans capable of such elaboration. This results in a smaller overall shift in molecular weight. Whether this could alter the function of pNAL remains an interesting issue that warrants further investigation and is beyond the scope of this study.

Comment: In Figure 1, I would perhaps consider leaving out the human pathway (A), as it does not directly pertain to the work presented.

Response:
Thank you for your suggestion. We would like to include the human pathway to highlight the important differences between these distantly related eukaryotes, such as the transfer of oligo/pauci-mannose in *Trypanosoma brucei* compared to the processing of oligomannose *N*-linked glycans in humans, and the different GT family repertoires, among others.

Comment: Lines 198-203: Do the authors mean that the iterative deletions would lead to shorter glycans, or that the mutants could produce all the glycans they describe?

Response: We propose that the double null mutants would be restricted to the *N*-glycan structures described, all of which should lack pNAL modification and are therefore restricted in size. For clarity, we have changed line 203 to include:

*"(Galβ1-4GlcNAcβ1-2)$_2$Man$_3$GlcNAc$_2$ complex N-glycans **devoid of pNAL** (Fig 4A), some of which…"*

Comment: Lines 252-259: What criteria were used to identify these proteins from the RCA pulldown? Could they do some tagging or something to directly show change in glyTs affects the MW of the proteins?

Response: The proteins were identified based on changes in electrophoretic mobility and confirmed by LC-MS/MS. We have clarified this further by modifying the text in line 254-255 to read:

*"and 6 and 7 predicted N-glycosylation sites, respectively [27] (S4 Fig) **indicative of Golgi trafficking and elaboration by glycosyltransferases**"*

Comment: Line 324: "EndoH", and "Endo H" are both used in the manuscript.

Response: Thank you for identifying this. We have standardised the usage to "Endo H" throughout the manuscript.

Comment: Line 324-326: This sentence is quite confusing, with the "unlike ESAG7" phrase being the main culprit. Consider revising, as it's very important for the interpretation of the rest of the figure.

Response: Thank you for this important point, we have revised the sentence and removed "unlike ESAG7" which makes the sentence clearer.

Comment: Line 336-341: Perhaps a quick explanation of why some bands are protected from Endo H treatment could be useful here for non-glyco experts.

Response: We have modified line 340 to read:

*"To investigate this further, we treated affinity-purified TfR with endo-β-N-acetylglycosidase H (Endo H) **which selectively removes oligomannose** N-glycans…"*

Comment: Fig 8E- I would include a scale bar for the micrographs.

Response: Included and figure legend updated.

Comment: Figure 8D-E. I think this data is contradictory. Unless the hypoglycosylated TfR is a significantly better binder of transferrin? Is there TfR on the cell surface, which could explain the decreased FP signal without seeing a loss in overall TfR signal on the WB?

Response: As shown in the new supplementary figure (S9 Fig), the data indicate that the anti-TfR signal is slightly reduced in the mutants, as mentioned in the text (lines 415-417), and remains localised within the flagellar pocket. As shown in the images, there is always some background surface signal in anti-TfR immunofluorescence images in all the cell lines, including WT. Whether the surface signal is merely noise or true signal, it is consistently present in all the cell lines. For this reason, we heavily relied on flow cytometry data for reliable quantitation of Alexa Fluor-conjugated transferrin fluorescence in uptake experiments, which measures functional endocytic capacity of TfR. The interesting and important question of whether pNAL is involved in flagellar pocket retention of TfR is therefore not supported by our data, and this question warrant further investigation.

Comment: Line 383: "wild type" not "wild"
Response: Fixed

Reviewer #2: (No Response).

Reviewer #3:

Comment: Is there any transcriptomics or PCR evidence that in the absence of TbGT8 or TbGT10, or both, there is an increase in the expression of other parasite GTs?

Response: Thank you for raising an interesting question. Unfortunately, we do not currently have transcriptomics or PCR data specifically addressing altered expression of other GTs in the absence of both TbGT8 and TbGT10. However, we (Duncan et al., 2021, PMCID: PMC8477195) previously reported increased activity of TbGT10 in TbGT8 knockout mutants, detected by Western blotting of whole cell lysates using an antibody (mAb139) that recognises -6Galβ1-4GlcNAcβ1- linkages of three repeats or more. Whether this increased activity results from elevated expression or reduced substrate competition is yet to be determined. We acknowledge that additional transcriptomic data could provide further insights, and we consider this an important area for future research.

Comment: Line 82: LLO abbreviation is repeated (line 75).
Response: Thank you for spotting this. Fixed

Comment: Line 215 and Fig. 4C: maybe I missed the point, but Fig 4C and Fig. 4B looks like the same experiment to me.

Response: You are correct that Figures 4B and 4C are similar; however, Figure 4B analyses the conditional null mutant, while Figure 4C examines the null mutant, selected as a stable gene knockout. The inclusion of both figures is intended to demonstrate that the glycosylation profile remains consistent even after the selection of a stable null mutant.

Comment: Line 259: please correct “endosmal”.
Response: Corrected. Thank you.

Line 290 and Fig. 5C: the authors claim by lectin blotting that the binding of TL is not affected in GT8 cells. By FC, however, they realize that the reduction is even greater in GT8 cells. Is there any statistical analysis backing this claim?

Response: Our claim that GT8 KO cells have reduced TL binding is supported by statistical analysis of TL binding using flow cytometry (FC). At 4°C, there is no statistically significant difference in TL binding between the conditional null mutant cells expressing TbGT8 and WT. However, when TbGT8 is ablated, the reduction in TL binding becomes significant, as confirmed by unpaired t-tests.

Comment: Line 336: it should be dESAG6, not dESAG.
Response: Fixed

Comment: Line 400: I think the authors refer to Fig. 8E, not to Fig. 8D.
Response: You are correct. Fixed.

Comment: Line 414 and Fig. 9: I found confusing the dextran explanation in the figure legend. Also, there’s no 1:1000 indication in the image, although it is mentioned in the text.

Response: We used chitin hydrolysate only at the highest concentration of 1:10 for Dextran as a control for fluid phase endocytosis, as mentioned in line 433 of the results section and line 1021 in the figure legend. This detail is also referred to in lines 489-491.

Comment: S2 Fig. has a very low-quality images, but this might be my own copy.

Response: We acknowledge that the quality of S2 Fig. was suboptimal in our copy as well. We have replaced it with a higher-quality version.
